# Oral squamous cell carcinoma: Effect of tobacco and alcohol on cancer location

**DOI:** 10.18332/tid/189303

**Published:** 2024-06-18

**Authors:** Riikka Eloranta, Suvi-Tuuli Vilén, Arvi Keinänen, Tuula Salo, Ahmed Qannam, Ibrahim O. Bello, Johanna Snäll

**Affiliations:** 1 Department of Oral and Maxillofacial Diseases, Clinicum, Faculty of Medicine, University of Helsinki, Helsinki University Hospital, Helsinki, Finland; 2 Department of Oral and Maxillofacial Diseases, Kymenlaakso Central Hospital Kotka, Kotka, Finland; 3 Translational Immunology Research Program (TRIMM), University of Helsinki, Helsinki, Finland; 4 Research Unit of Population Health, Faculty of Medicine, University of Oulu, Oulu, Finland; 5 Medical Research Center, Oulu University Hospital, University of Oulu, Oulu, Finland; 6 Department of Pathology, Helsinki University Hospital, University of Helsinki, Helsinki, Finland; 7 Department of Oral Medicine and Diagnostic Sciences, King Saud University College of Dentistry, Riyadh, Saudi Arabia

**Keywords:** oral cancer, squamous cell cancer, carcinogen, alcohol, smoking, tumor site

## Abstract

**INTRODUCTION:**

The underlying factors of oral squamous cell cancers (OSCC) have been elucidated, but studies have focused little on etiological differences in affected oral cavity sites. The aim of this retrospective study was to clarify the role of carcinogen exposure in OSCC of different oral cavity areas.

**METHODS:**

A cross-sectional study of patients with primary OSCC was conducted retrospectively, based on patient records from Helsinki University Hospital, Finland, between January 2016 and December 2020. The patients’ self-reported history of tobacco smoking and alcohol use was explained by tumor site, age, sex, tumor size, and lymph node status in a logistic regression model. The information on smoking and alcohol use was compiled from a patient background form.

**RESULTS:**

In 519 patients, tumors occurred most often in the tongue (51%), gingiva (21%), or floor of the mouth (FOM; 15%). FOM had 26-fold greater odds for a history of smoking and alcohol use than other tumor sites (OR=25.78; 95% CI: 8.02–82.95; p<0.001). Gingival and buccal sites were associated significantly less with smoking and alcohol use (OR=0.43, 95% CI: 0.28–0.67; p<0.001 and OR=0.47; 95% CI: 0.25–0.92; p<0.026, respectively). Patients of older age were less likely to have a history of smoking and alcohol use (AOR=0.95; 95% CI: 0.94–0.97; p<0.001) than younger patients. Tumor size (T3-4) and FOM increased the odds for history of smoking and alcohol use (AOR=1.73; 95% CI: 1.15–2.60; p=0.009 and AOR=26.15; 95% CI: 8.01–84.84; p<0.001, respectively).

**CONCLUSIONS:**

OSCC of oral cavity sites has notable differences in etiology. FOM seems to be related almost exclusively to conventional smoking and heavy alcohol use.

## INTRODUCTION

Alcohol and tobacco are the most recognized risk factors for oral squamous cell carcinoma (OSCC)^1^. Alcohol acts mostly through its metabolic product acetaldehyde, which can bind to DNA and form genotoxic DNA adducts. These can induce DNA point mutations, double-strand breaks, and other structural changes in the genome^2^. Tobacco, in turn, acts via tobacco-specific nitrosamines (TSNAs) and other carcinogens^3^. Metabolized N'-nitrosonornicotine (NNN) forms highly reactive molecules, potentially resulting in DNA adducts that can cause miscoding in DNA replication^4^. Tobacco-specific nitrosamines are formed by combustion, chief among which are polycyclic aromatic hydrocarbons (PAHs). Other combustion products in cigarette smoke are, for example, acetaldehyde and acrolein^3^.

The type of alcohol or tobacco is related to the risk, with especially black tobacco and spirits being associated with OSCC development^5^. The combined use of both increases the risk of OSCC by 15-fold, especially for floor-of-mouth (FOM) cancer^1^. Compared with regular cigarettes, pipes and cigars seem to have a significantly higher risk for OSCC, probably due to the alkaline smoke that tends to stay longer in the mouth^5^. The risk of OSCC increases with the daily quantity of carcinogenic products, duration of consumption^6^, and lifetime cumulative consumption of both alcohol and tobacco^7^.

There are other important risk factors as well, since especially the incidence of oral tongue squamous cell carcinoma (OTSCC) has been rising in young non-smoking, and non-drinking female patients^8^. Betel quid chewing, diet, family history of cancer, oral and dental health issues, ill-fitting dentures, mechanical irritation, oral lichen planus, lichenoid reactions, immunosuppression, and vitamin/nutrition deficiencies have been reported as predisposing factors for OSCC^9,10^. Human papillomavirus (HPV) is not associated with OTSCC^11^ but is related to cancers of the base of the tongue and the oropharynx. These other risk factors seem to be more common in never-smoking, never-drinking patients^8^.

Local friction, mucosal thickness, and how tightly bound the mucosa is to the underlying structure may affect cancer development^12^. OSCC development is associated with HPV and potentially with other viruses or microbes^8^, but the suggested role of various carcinogenic factors in specific OSCC sites varies between studies. Some studies have found that FOM and retromolar trigone are the most susceptible to carcinogens^13,14^, while others suggest that the risk is higher for the tongue. The risk of tobacco and alcohol is connected to local exposure. This is attributed to pooling the carcinogens with saliva^12^, which predisposes the thin and non-keratinized mucosa of FOM to repeated exposure to carcinogens^15^. Conversely, gingival OSCC seems rarer among smokers, perhaps because the keratin on the attached gingiva protects the gingiva from carcinogens^16^.

The purpose of this study was to clarify the role of tobacco smoking and alcohol in different OSCC sites. Our hypothesis was that the role of preceding smoking and alcohol exposure varies according to the site of oral cancer.

## METHODS

### Patient material

A cross-sectional study of patients with primary OSCC was conducted retrospectively. Data of all OSCC patients with primary OSCC evaluated at Helsinki University Hospital, Helsinki, Finland, between January 2016 and December 2020 were examined retrospectively. Patient data were retrieved from the multidisciplinary Head and Neck Tumor Board of Helsinki University Hospital.

As supplementary data of comparative geographical and lifestyle effect, descriptive statistics of patients diagnosed with OSCC during the same period (from 2016 to 2020) at King Saud University Oral Pathology Lab, Riyadh, Saudi Arabia, are presented.

### Inclusion and exclusion criteria

All patients with a primary OSCC diagnosis evaluated during the study period were included. Patients with a history of previous oral cavity cancer were excluded.

### Study design

The information on tobacco smoking and alcohol use was compiled from a separate background data form that all cancer patients fill in. The association between patients’ self-reported history of smoking and alcohol use and the location of OSCC was investigated. Tumor sites were grouped as tongue, buccal mucosa, gingiva, palate, and FOM. In addition, an anamnestic self-reported history of smoking and alcohol use was analyzed in more detail in patients with OSCC of FOM. For the Saudi Arabian cases, the data on tobacco and alcohol use were obtained from the laboratory request forms filled from the patient files of the Oral and Maxillofacial Surgery Clinic of KSU Dental University Hospital.

The self-reported information on smoking and alcohol use was compiled from a patient background form that all cancer patients fill in in the hospital. Patients were grouped by smoking habit into two groups: non-smokers (non-smokers, and former smokers in cessation ≥5 years) and smokers (current smokers, and former smokers in cessation <5 years). Occasional smokers were included as non-smokers. Regarding alcohol, seven doses per week or more was defined as heavy alcohol use, as alcohol consumption of 70 g per week or more is estimated to increase the risk of cancer^17^. One dose corresponds to 10–12 g of pure alcohol.

Tumor sites were grouped as tongue, buccal mucosa, gingiva, palate, and FOM according to The International Classification of Diseases 10th codes. Other variables investigated were age, sex, tumor size, and lymph node status. Tumor size was defined according to T categorization as T1-4 based on TNM Staging of Lip and Oral Cavity cancers – AJCC 7th Edition^18^ and 8th Edition^19,20^ valid at the time of diagnosis. Pathological lymph node status was categorized as N0 and N1 or more.

### Statistical analysis

The self-reported history of tobacco smoking and heavy alcohol use (either or both) was explained with patients’ clinical data in a logistic regression analysis. Logistic regression analyses were evaluated for goodness-of-fit using the Hosmer and Lemeshow test. Based on the previous literature, patient demographics, tumor size, and lymph node metastasis were chosen as covariates in a multivariate model. Prior to conducting multiple logistic regression analyses, binary logistic regression analyses were conducted to detect possible multicollinearity among categorical explanatory variables. Sex and lymph node metastasis were omitted due to a strong association with age and T-classification, respectively. A significance level of 0.05 was set for all analyses. Statistical analyses were performed using SPSS 28.0 (IBM Corp., Armonk, NY, USA).

### Ethical approval

The study was approved by the Internal Review Board of the Head and Neck Centre, Helsinki University Hospital, Helsinki, Finland (HUS/66/2018) and the KSU College of Dentistry Research Center (CDRC/FR 0264).

## RESULTS

In all, 519 patients with OSCC fulfilled the inclusion criteria and were included in the final analyses. Patient age ranged between 19 and 98 years (mean = 66.3 years). OSCC patients were more often male (59%) than female (41%) (Table 1). More than half (57%) of the patients reported a history of either smoking, heavy alcohol use, or both. Half (50.5%) of the patients were smokers (current smokers, and former smokers in cessation <5 years). Heavy alcohol consumption was recorded in 38% of all OSCCs. Altogether, 202 (39%) of all 519 patients had tumors that belonged to the TNM classification T1 category.

**Table 1:** Descriptive statistics of oral squamous cell carcinoma patients, Helsinki University Hospital, Finland, January 2016 – December 2020 (N=519)

*Characteristics*	*n*	*%*
**Sex**		
Male	306	59.0
Female	213	41.0
**Age** (years)		
Range	19 – 98	
Mean	66.3	
Median	66.0	
**Smoking and heavy alcohol use** (either or both)		
Yes	296	57.0
No	223	43.0
Smoking*		
Yes	262	50.5
No	257	49.5
**Alcohol consumption 7 portions/week**		
Yes	198	38.2
No	321	61.8
**T-classification**		
T1	202	38.9
T2	132	25.4
T3	71	13.7
T4	114	22.0
**Lymph node metastasis**		
Yes	155	29.9
No	364	70.1

* Current smoking or cessation <5 years ago

**Figure 1:**
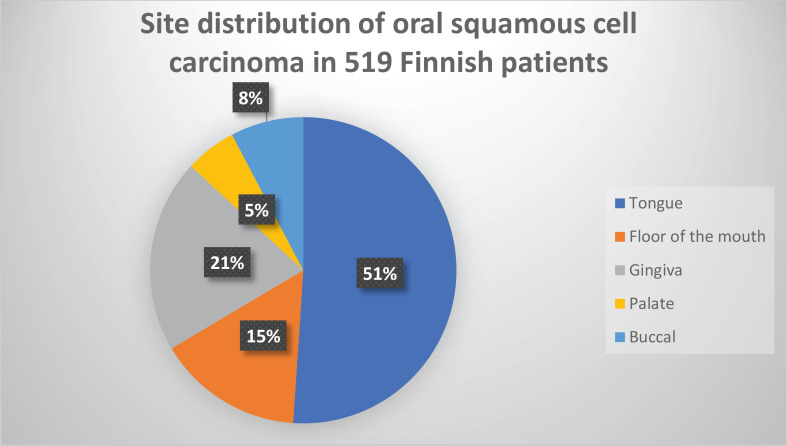
Oral squamous cell was found to be most often located in the tongue (51%). The second most common location was the gingiva (21%), followed by the floor of the mouth (15%)

The most common site of OSCC was the tongue (51%), followed by gingiva (21%) and FOM (15%). Less common sites were buccal mucosa (8%) and palate (5%) (Figure 1).

**Figure 2:**
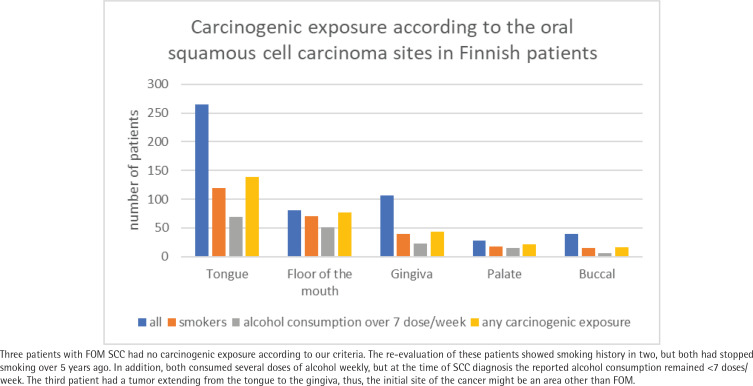
Carcinogenic exposure and history of smoking and alcohol consumption varied between tumor sites

History of self-reported smoking and alcohol use differed between OSCC sites (Figure 2). Only half (52%) of the patients with OSCC reported smoking and heavy alcohol use (either or both). In contrast, almost all patients (96%) with FOM squamous cell carcinoma (SCC) had had prior exposure to those. Patients with FOM SCC were 26-fold more likely to have such a history of anamnesis than other tumor sites (OR=25.78; 95% CI: 8.02–82.95; p<0.001). By contrast, patients with gingival SCC had significantly less exposure to tobacco and alcohol in their history (OR=0.43; 95% CI: 0.28–0.67; p<0.001) (Table 2). Patients with OSCC of buccal mucosa and tongue were less likely to have preceding smoking and alcohol exposure as well (OR=0.47; 95% CI: 0.25–0.92; p<0.026 and OR=0.68; 95% CI: 0.48–0.97; p<0.032, respectively).

**Table 2:** Univariate logistic regression model explaining self-reported history of smoking and heavy alcohol use* among patients with oral squamous cell carcinoma sites, Helsinki University Hospital, Finland, January 2016 – December 2020 (N=519)

*Variable*	*OR*	*95% CI*	*p*
Tongue (Ref. other)	0.68	0.48–0.97	**0.032**
Gingiva (Ref. other)	0.43	0.28–0.67	**<0.001**
Floor of the mouth (Ref. other)	25.78	8.02–82.95	**<0.001**
Palatinum (Ref. other)	3.70	0.98–5.65	0.055
Buccal (Ref. other)	0.47	0.25–0.92	**0.026**

* Either or both.

Univariate logistic regression analyses revealed associations of age, sex, and lymph node metastases with a history of smoking or heavy alcohol use (Table 3). Older patients had significantly less likely OSCC associated with a history of smoking and alcohol use than younger patients (OR=0.95; 95% CI: 0.94–0.97; p<0.001). Patients with lymph node metastases had more than two times greater odds for self-reported history of smoking and alcohol use than patients without lymph node metastases (OR=2.15; 95% CI: 1.44–3.21; p<0.001). A multivariate analysis was conducted to explain exposure with site adjustments for age and T-classification. Age is associated significantly with a history of smoking and alcohol use (AOR=0.95; 95% CI: 0.94–0.97; p<0.001) (Table 3). Moreover, larger tumor size and FOM increased the odds for smoking and alcohol use independently (AOR=1.73; 95% CI: 1.15–2.60; p=0.009 and AOR=26.15; 95% CI: 8.01–84.84; p<0.001, respectively).

**Table 3:** Logistic regression model explaining the self-reported history of smoking and heavy alcohol use* with patient demographics, tumor size, and lymph node metastasis among patients with oral squamous cell carcinoma sites, Helsinki University Hospital, Finland, January 2016 – December 2020 (N=519)

*Univariate logistic regression analysis*				Multivariate logistic regression analysis**			
*Variable*	*OR*	*95% CI*	*p*	*Variable*	*AOR*	*95% CI*	*p*
Age (years)	0.95	0.94–0.97	**<0.001**	Age (years)	0.95	0.94–0.97	**<0.001**
Sex (Ref. male)	0.33	0.23–0.48	**<0.001**	T-classification (Ref. T1-T2)	1.73	1.15–2.60	**0.009**
T-classification (Ref. T1-T2)	1.25	0.87–1.80	0.230	Floor of the mouth (Ref. no)	26.15	8.01–84.84	**<0.001**
Lymph node metastasis (Ref. no)	2.15	1.44–3.21	**<0.001**				
Floor of the mouth (Ref. no)	25.78	8.02–82.95	**<0.001**				

* Either or both. AOR: adjusted odds ratio.

** Multivariate analysis was adjusted with age and T-classification. Sex and lymph node metastasis were omitted because of strong association with age and T-classification, respectively.

Further univariate logistic regression analyses showed statistical associations of sex and preceding history of smoking or alcohol use with FOM. Males were 2.5 times more likely to have SCC of FOM than females (OR=2.54; 95% CI: 1.47–4.40; p<0.001) (Table 4). Smoking and heavy alcohol use increased the odds for SCC of FOM significantly (OR=9.01; 95% CI: 4.52–17.93; p<0.001 and OR=5.63; 95% CI: 3.31–9.57; p<0.001, respectively). Multivariate logistic regression analysis for smoking and alcohol exposure could not be performed due to intercorrelations of the variables.

**Table 4:** Univariate logistic regression model for explaining the presence of floor-of-the-mouth squamous cell carcinoma with patient demographics, tumor size, lymph node metastasis, and self-reported history of smoking and heavy alcohol use among patients with oral squamous cell carcinoma sites, Helsinki University Hospital, Finland, January 2016 – December 2020 (N=519)

*Variable*	*OR*	*95% CI*	*p*
Age (years)	0.98	0.97–1.00	0.090
Sex (Ref. female)	2.54	1.47–4.40	**<0.001**
T-classification (Ref. T1-T2)	0.60	0.35–1.02	0.058
Lymph node metastasis (Ref. no)	1.61	0.98–2.64	0.061
Smoking and heavy alcohol use* (Ref. no)	25.78	8.02–82.95	**<0.001**
Smoking (Ref. no)	9.01	4.52–17.93	**<0.001**
Heavy alcohol use (Ref. no)	5.63	3.31–9.57	**<0.001**

* Either or both.

In the Supplementary file concerning Saudi-Arabian patients, site distribution differs notably from the previous data (Supplementary file Figure 1). Lack of exposure to common carcinogens, particularly alcohol, explains the difference between Finnish and Saudi-Arabian populations (Supplementary file Figure 2).

## DISCUSSION

In this study, we clarified the role of tobacco smoking and alcohol use in different OSCC sites in a retrospective cohort study. In all, 57% of the Finnish OSCC patients had a history of carcinogenic exposure. Our hypothesis was confirmed, as preceding carcinogen exposure was strongly associated with specific OSCC sites. Of the patients with FOM SCC, 98% had notable prior carcinogenic exposure. Carcinogen exposure also seemed to be significant in patients with SCC of the palate, whereas, in patients with tongue, buccal, and gingival SCC, those without carcinogen exposure were more represented (Figure 2).

OSCC is the most common malignancy of the oral cavity, and it can affect any site intraorally^21^. The tongue was the most common site in this study, which aligns with earlier studies^22^. Conversely, the gingiva being the second, the FOM the third, and the buccal mucosa only the fourth differ from recent studies in other populations^22^. Discrepancies between the studies are most likely explained by differences in study populations, especially regarding smoking and heavy alcohol exposure. In our study, patients’ alcohol and smoking use was common; 57% of patients reported either smoking or alcohol use or both. This increases the proportion of FOM. As shown in this study, smoking and alcohol exposure was 25.8-fold more likely to be related to SCC of FOM than to other OSCC sites (Table 4). Other investigators have previously highlighted the association^1,23^, but the mechanism underlying the differences remains obscure^13,14,16^.

The structure of FOM could explain why it is more sensitive to carcinogens. The oral cavity sites differ in tissue composition, molecular marker expression, and epithelial turnover rates^21^. FOM has thin, non-keratinized mucosa, and the carcinogens tend to pool when mixed with saliva, allowing more contact time with noxious substances^16^. Non-keratinized epithelium has a turnover rate of 25 days, and keratinized epithelium about 50 days^24^. The keratin surface acts as a barrier, protecting the mucosa against the environment^25^. Since FOM lacks this barrier, it is more sensitive to carcinogens and the heat of tobacco smoke. Whereas the OSCCs of the non-keratinized lining epithelium of the FOM and buccal mucosa have been linked to tobacco and alcohol consumption, the OSCCs originating from the keratinized masticatory epithelium of the gingiva and hard palate are more commonly seen in women with no risk behavior^26^. The structural differences could explain why FOM is more sensitive to carcinogens and, therefore, seen more frequently in smoking and drinking patients. However, since there has been a rise in non-smoking non-drinking (NSND) patients with tongue cancer, the question is what factors not affecting FOM could explain the increase in OTSCC cases.

In the present study population, only three out of 80 patients with FOM OSCC were NSND. Further evaluation showed moderate carcinogenic exposure in the recent history of two of these patients. The comparison is interesting, especially for OSCC and FOM (Figure 2). There have been suggestions for causal factors of OTSCC, such as impaired immune system^9^ and genetic origin (Li-Fraumeni syndrome, Fanconi’s anemia)^27^. Dysbiotic oral microbiome, apart from HPV, has been considered a predisposing factor in OTSCC^28^, but findings have remained unclear^29^. FOM carcinomas usually also have a low incidence of HPV-DNA, and HPV does not seem to have a significant role in the pathogenesis of FOM OSCC^30^. Among the young, non-smoking patients with OTSCC, a significantly higher prevalence of oral leukoplakia has been identified^31^, which is considered a stronger risk factor for non-smokers than for smokers^32^. The tongue is the most common or the second most common site of oral leukoplakia (following buccal mucosa) and is considered a high-risk site for malignant transformation^28^. The differences in the etiology of these cancers remain partly unknown, but examining whether FOM has structural and/or molecular differences from the tongue that could attenuate the effect of these risk factors should be elucidated.

The molecular expression differences in OSCCs have been studied for different oral cavity sites. At least buccal mucosa and OTSCCs have significantly different expressions of p16 and p21 since downregulation of p16 and p21 expression was seen in 47% of OTSCC cases and only 28% of buccal mucosa carcinomas^21,33^. Telomerase activity has also been reported to differ significantly among tumors in non-keratinized mucosa like FOM and ventrolateral tongue^34^. Keratinized and non-keratinized epithelia also differ in cellular protein content^26^. A study examining the site-specific gene expression of OSCC found differences in protein regulation patterns, especially for tongue and FOM OSCCs. XIAP, an inhibitor of apoptosis that prevents caspase activation, was strongly expressed in FOM SCCs, in contrast to OTSCCs, in which the expression levels were low^35,36^. Similar variable results were also seen in expression levels of p53, CA IX, beta-catenin, HIf-1-alpha, and c-kit among these sites^36^.

### Limitations

The retrospective setting of the study has some limitations. Smoking and alcohol history was based on patient self-report, which likely leads to underreporting of results. Therefore, the limit of heavy alcohol consumption was set at seven servings per week, according to a previous cancer study^37^. Different tobacco products could not be determined. In addition, the number of cigarettes was not assessed. Our findings also have limited generalizability to other countries. At the population level, the differences can be explained by the effects of carcinogenic sources other than tobacco, smoking, and alcohol.

OSCCs of different sites have differences in etiology. SCC of FOM appears to be almost entirely cancer caused by the common carcinogens of tobacco and alcohol. Examining the site-wise heterogeneity in OSCC would also be important for planning cancer treatment since tumor sites seem to differ in molecular expression profiles. FOM may be deficient in some structures that predispose to OTSCC, the rate of which has increased in NSND patients. In addition, the male-to-female ratio is also higher for FOM cancer than for cancers of the gingiva or tongue. Although differences in alcohol use and smoking habits could partly explain this, gender-associated endogenous factors also warrant investigation^16^.

## CONCLUSIONS

OSCC of different sites should be considered as different entities to find preventive and predisposing factors for malignancy. In the future, OSCC treatments may differ according to etiology.

## Supplementary Material



## Data Availability

The data supporting this research are available from the authors on reasonable request.
