# Supplementary material for: Oral squamous cell carcinoma: Effect of tobacco and alcohol on cancer location
Source: Tob Induc Dis. 2024 Jun 18;22:10.18332/tid/189303. doi: 10.18332/tid/189303 (PMC11185050; doi:10.18332/tid/189303)
Supplement: Supplementary file 1 [file TID-22-112-s1.pdf]

Supplementary Figure 1. Tumor site of oral squamous cell cancer in Saudi-Arabian patients differed from the corresponding Finnish population (Figure 1 in main text). Squamous cell cancer of the floor of the mouth was almost completely absent in Saudi-Arabian patients

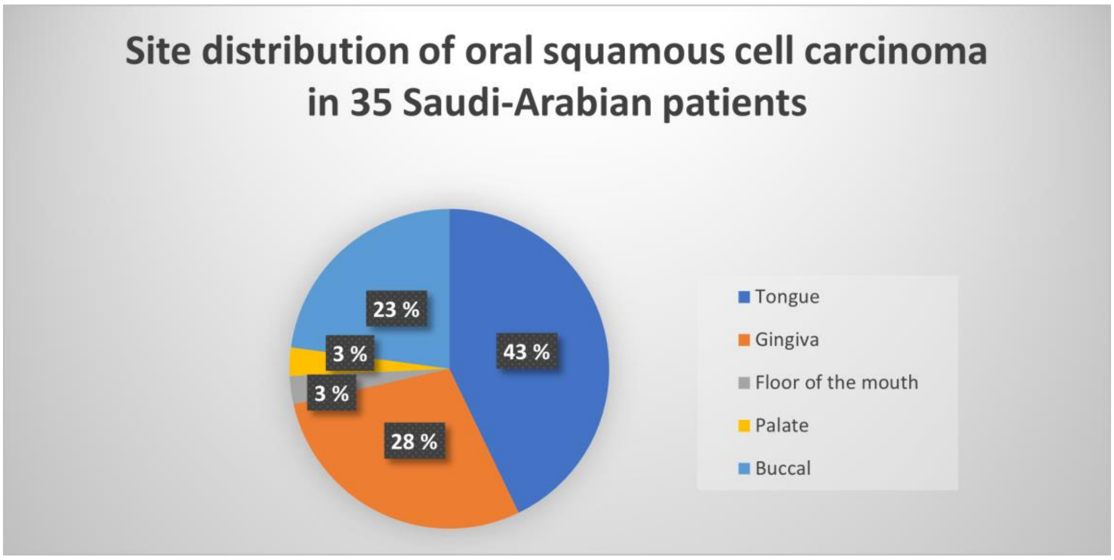

Supplementary Figure 2. Lack of smoking and heavy alcohol use in Saudi-Arabian patients explains the difference between Saudi-Arabian and Finnish populations (Figure 2 in main text).

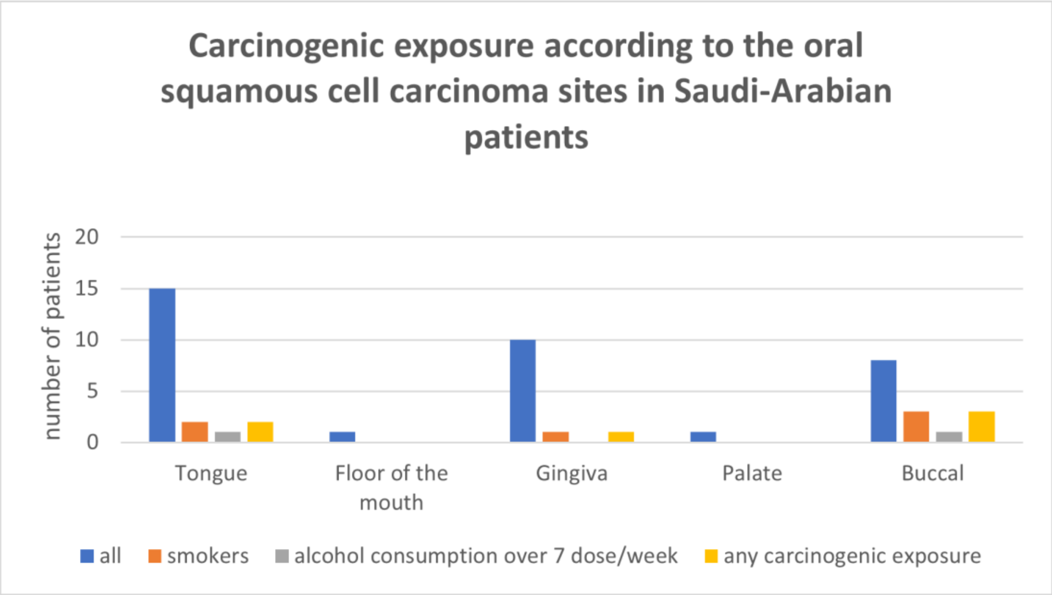

© 2024 Eloranta R. et al.
